# Immunosenescence and multiple sclerosis: inflammaging for prognosis and therapeutic consideration

**DOI:** 10.3389/fragi.2023.1234572

**Published:** 2023-10-13

**Authors:** Smathorn Thakolwiboon, Elizabeth A. Mills, Jennifer Yang, Jonathan Doty, Martin I. Belkin, Thomas Cho, Charles Schultz, Yang Mao-Draayer

**Affiliations:** ^1^ Department of Neurology, University of Michigan, Ann Arbor, MI, United States; ^2^ Michigan Institute for Neurological Disorders, Farmington Hills, MI, United States; ^3^ Autoimmune Center of Excellence, University of Michigan, Ann Arbor, MI, United States; ^4^ Graduate Program in Immunology, Program in Biomedical Sciences, University of Michigan, Ann Arbor, MI, United States

**Keywords:** multiple sclerosis, immunosenescence, inflammaging, neuroinflammation, neurodegeneration

## Abstract

Aging is associated with a progressive decline of innate and adaptive immune responses, called immunosenescence. This phenomenon links to different multiple sclerosis (MS) disease courses among different age groups. While clinical relapse and active demyelination are mainly related to the altered adaptive immunity, including invasion of T- and B-lymphocytes, impairment of innate immune cell (e.g., microglia, astrocyte) function is the main contributor to disability progression and neurodegeneration. Most patients with MS manifest the relapsing-remitting phenotype at a younger age, while progressive phenotypes are mainly seen in older patients. Current disease-modifying therapies (DMTs) primarily targeting adaptive immunity are less efficacious in older patients, suggesting that immunosenescence plays a role in treatment response. This review summarizes the recent immune mechanistic studies regarding immunosenescence in patients with MS and discusses the clinical implications of these findings.

## 1 Introduction

Traditionally, multiple sclerosis (MS) is considered an immune-mediated inflammatory demyelinating disease of the central nervous system (CNS). However, it is well known that neurodegenerative processes are also involved and play a key role in cumulative disability. While current disease-modifying therapies (DMTs) effectively control inflammation, they do not fully stop disability progression, possibly due to inadequate targeting of neurodegeneration. The lack of clarity regarding the etiology of progression hinders the development of effective therapies for progressive disease. Immunosenescence has emerged as a possible mechanism (Dema et al., 2021).

Immunosenescence is an age-related weakening of adaptative and innate immune responses with altered activation (Nikolich-Žugich, 2018). As a result, older individuals may clear pathogens less effectively, resulting in chronic low-grade inflammation, known as inflammaging, which contributes to tissue damage and neurodegenerative diseases. A multifaceted etiology of inflammaging has been suggested including reduced diversity of gut microbiota, genetic polymorphisms, and obesity, in addition to immunosenescence (Ferrucci and Fabbri, 2018).

Some studies have demonstrated premature immunosenescence in MS (Haegert et al., 2011) and experimental autoimmune encephalitis (EAE) in mice (Zeydan and Kantarci, 2020). Immune system changes have a significant impact on the MS disease course, risk of infection, vaccine efficacy, and response to treatment (Grebenciucova and Berger, 2017). Due to longevity and DMT availability, the number of older patients with MS is increasing rapidly. Though 20 to 40 is the average age of MS onset, 10% develop the disease after age 50, known as late-onset MS (LOMS). Patients with LOMS have a higher proportion of primary progressive MS (PPMS) and a faster rate of progressive disability when compared to those who develop MS before age 50. However, it is interesting to note that the same study found that patients who developed disease prior to the age of 50 had a higher risk of reaching Expanded Disability Status Scale (EDSS) 6 (Andersen et al., 2021). This yields consideration that age-related immune changes may play a role in disease progression. Consequently, understanding these changes in a setting of immunosenescence is crucial to the care of patients with MS in an aging world.

**FIGURE 1:**
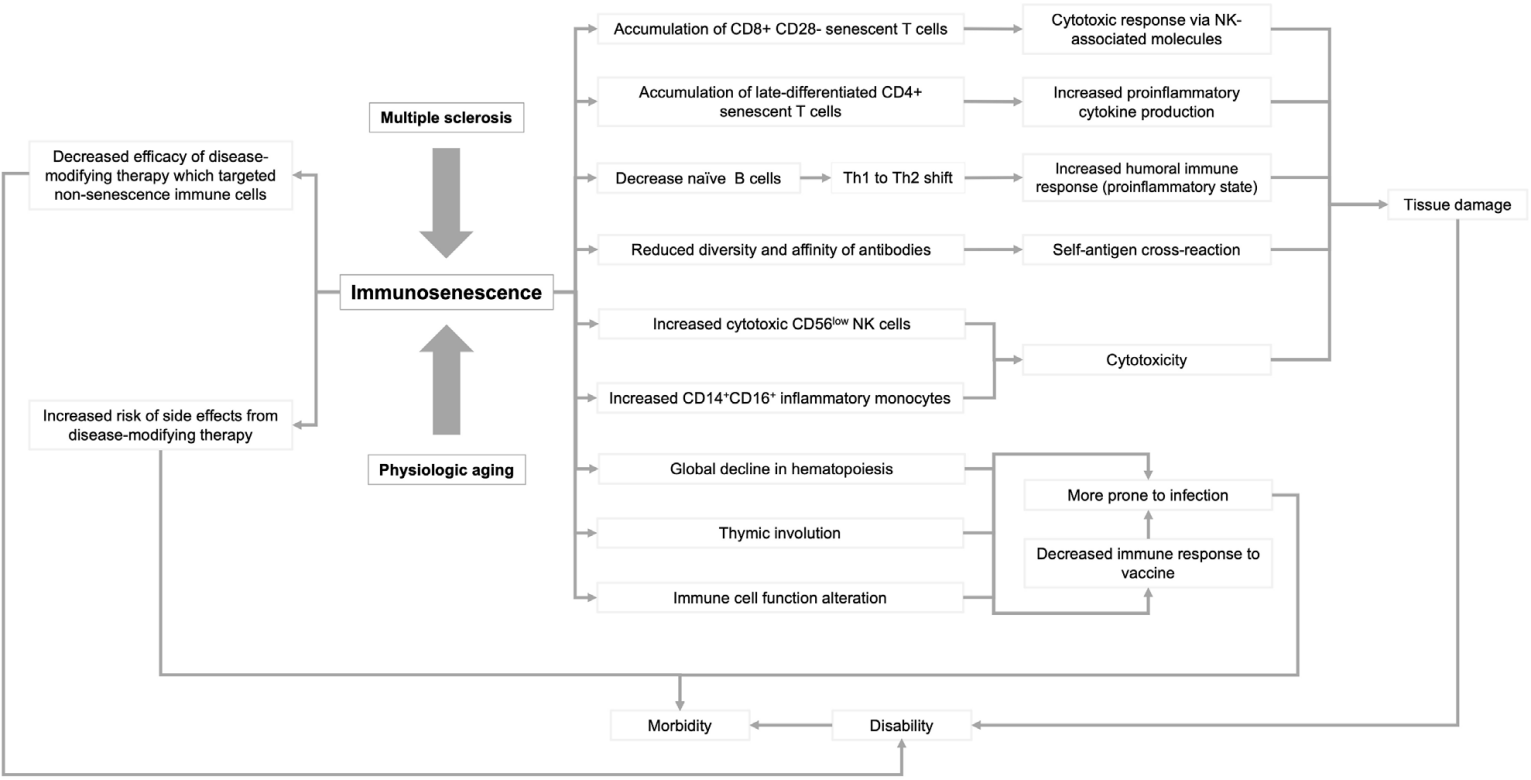
Influence of multiple sclerosis and physiological aging on immunosenescence. Immune dysfunction that occurs as a result of aging and multiple sclerosis can lead to a variety of physiological responses that can ultimately lead to disability and morbidity. Arrowheads indicate the directionality of the sequence of events.

In this review, we summarize the current knowledge of immunosenescence, focusing on humans (Figure 1). We then discuss age-related changes in the adaptive, innate, and CNS immune systems. Finally, we discuss immunosenescence in patients with MS and the clinical implications of this emerging knowledge.

## 2 Aging and immune system changes

Immune cells are generated from hematopoietic stem cells (HSCs) in the bone marrow. They stepwise differentiate and undergo selection and proliferation upon antigenic exposure; thus, becoming prone to the senescence process. Telomere shortening and cell cycle arrest in aging HSCs result in fewer immune cells (Rossi et al., 2005; Vaiserman and Krasnienkov, 2020). Senescent cells can undergo changes to their secretory profile, increasing soluble proteases or insoluble extracellular proteins, which contribute to a pro-inflammatory milieu known as the senescence-associated secretory phenotype (SASP). (Adler et al., 2007; Coppé et al., 2010; Muñoz-Espín and Serrano, 2014). A combination of accumulation of cell debris, self-antigens, and the SASP leads to inflammaging. Immunosenescence impairs the immune response to infection and vaccines due to reduced antigenic response by T- and B-cells (Castle, , 2000; Frasca et al., 2016), contributing to cancer risk (Lian et al., 2020) and immune-mediated disorders in the elderly (Manoussakis et al., 1987; Ramos-Casals et al., 2003).

### 2.1 Adaptive immune system

Progressive thymus gland involution reduces the naïve T-cell pool and diversity of the T-cell receptor (TCR) repertoire. Reactive dendritic and B-cell homeostatic proliferation then leads to clonal expansion of memory T-cells, further depleting the TCR repertoire (Naylor et al., 2005; Pfister et al., 2006; Qi et al., 2014).

T-cell senescence results in a decrease in CD4^+^ T-cells and increase in CD8^+^ T-cells, causing an inversion of the CD4^+^/CD8^+^ ratio (Olsso et al., 2000). Late-differentiated memory CD8^+^ T-cells become senescent, characterized by CD28 loss, telomere shortening, and resistance to apoptosis (Derhovanessian et al., 2011). These senescent T-cells express CD57, killer-cell lectin-like receptor G1 (KLRG1), and natural killer (NK)-associated receptors NKG2D (Vallejo, 2005) and acquire a cytotoxic response via NK-associated molecules while losing response to TCR-mediated signals (Pereira et al., 2020). CD8^+^ T cells display SASP regulated by p38 MAPK signaling (Callender et al., 2018). Late-differentiated CD4^+^T-cells feature decreased CD28, increased NKG2D expression, elevated interferon-gamma (IFN-γ) production (Weyand et al., 1998; Alonso-Arias et al., 2011; Dema et al., 2021), and a lack of CD40 ligand expression. A shift from Th1 to Th2 cells and reduced IL-2 production were observed with aging (Shearer, 1997; Ginaldi et al., 1999a; Ginaldi et al., 1999b).

The percentage of total B-cells, including B-cell activating factor (BAFF) and A-proliferation-inducing ligand (APRIL), abruptly decreases after age 75 due to hematopoiesis. (Jin et al., 2008; Bulati et al., 2011). Progenitor B-cells, B-cell lymphopoiesis, and absolute count of relative peripheral CD19^+^ B-cells decrease with age, while proportions of B-cell subsets remain independent of age (Perdaens and van Pesch, 2021). The memory B-cell population expands with age (Frasc et al., 2017; Frasca et al., 2017). Naïve mature (IgD^+^CD27^−^) B-cells decrease while exhausted double-negative (IgD^−^CD27^−^) B-cells increase (Colonna-Romano et al., 2009) and produce higher levels of pro-inflammatory cytokines, including tumor necrosis factor-alpha (TNF-α), IL-6, and IL-8 in the elderly compared to younger individuals (Frasc et al., 2017). Immature transitional immunoregulatory CD24^high^CD38^high^ B-cells reduce with age, causing less IL-10 production (Duggal et al., 2013).

Serum IgM and IgD decrease with age, while concentrations of IgG and IgA increase due to a reduction in antibody class switching and affinity maturation in B-cells during clonal expansion (Listì et al., 2006; Frasca et al., 2008). Although B-cell antigen receptor repertoire diversity and response in the peripheral blood and lymph nodes are reduced in the elderly, somatic hypermutation does not change with age (Tabibian-Keissar et al., 2016).

Age-associated B-cells (ABCs), including IgD^−^CD27^−^ (aka. double negative B-cell) and CD21^−^CD11c^+^ (aka. CD21^low^ B-cell) (Hao et al., 2011; Rubtsov et al., 2011) are a subset of B-cells that increase with age, are pro-inflammatory, and prematurely increase with immune-mediated diseases (Ca and ncro, 2020). However, a recent study demonstrated a decline in B-cell population and inactivation of B-cell specific loci in men over 65 (Márquez et al., 2020).

### 2.2 Innate immune system

Aging affects the innate immune response through impaired pathogen associated molecular patterns (PAMPs)-mediated response, decreased type I interferon production, and fewer plasmacytoid dendritic cells (Molony et al., 2017; Feng et al., 2021). Monocyte number remains stable in the elderly (Seidler et al., 2010; Ratts and Weng, 2012), but there is an increase in CD14^+^CD16^+^ inflammatory monocytes (Nyugen et al., 2010; Seidler et al., 2010; Hearps et al., 2012). These aging monocytes produce less IFN-α, IFN-γ, IL-1β, CCL8, and CCL20 and express CX3C chemokine receptor-1 (CX3CR1), which plays a crucial role in monocyte survival (Metcalf et al., 2017). Moreover, they also overexpressed Tyro 3, Axl, and Mer (TAM) receptors, resulting in impaired clearance of apoptotic cells contributing to inflammation (Wang et al., 2018) and increased TLR4-and TLR8-dependent cytokine production.

Immunoregulatory CD56^high^ NK cells, which augment cytokine and chemokine production, decrease with aging while cytotoxic CD56^low^ NK cells increase (Caligiuri, 2008). NK cells also have defective granulation capacity (Borrego et al., 1999; Le Garff-Tavernier et al., 2010). Neutrophils function in the elderly has diminished suppressor of cytokine signaling 1 (SOCS1) and SOCS3 expression, dysregulation of the JAK-STAT pathway (Fo et al., 2007), and a defective response to triggering receptor expressed on myeloid cells 1 (TREM1), which is responsible for regulating cytokines, chemokines, and reactive oxygen species (ROS) production (Fortin et al., 2006). This can lead to apoptosis dysfunction, limited ROS generation, respiratory burst impairment, and altered cell surface molecule expression, and increased susceptibility to microbial infections (Butcher et al., 2001; Hazeldine et al., 2014; Lopes et al., 2018).

### 2.3 CNS immunity

Microglia and astrocytes are regulators of the innate and adaptive immune responses in the CNS. Microglia in the aging brain progressively lose their homeostatic molecular signature by developing increased production of pro-inflammatory cytokines, ROS, and dysfunctional lysosomal deposits, which are all linked to the pathogenesis of a growing number of neurodegenerative diseases (Angelova and Brown, 2019). These aged microglia contribute to recruitment of T cells into the CNS via increased TNF-α, which upregulates the adhesion molecules VCAM-1 and ICAM-1 (Zhang et al., 2022). Additionally, a subset of aged human microglia expresses high amounts of ferritin, which may be associated with dystrophic change and senescence, due to iron accumulation and dyshomeostasis, (Lopes et al., 2008; Ward et al., 2014) and may be a consequence of iron-containing oligodendrocyte destruction.

Senescent astrocytes lose homeostatic glutamate uptake, exhibit a propensity for pro-inflammatory responses, and increased expression of glial fibrillary acidic protein (GFAP) and vimentin (Sloane et al., 2000; Porchet et al., 2003) via transforming growth factor-beta 1 (TGFβ1) signaling. TGFβ1 inhibits astrocyte proliferation and induces SASP by increasing pro-inflammatory molecule expression (Coppé et al., 2010). Clearance of senescent astrocytes may also be impaired as age-related thickening of meningeal-lymphatic vessels has been observed in humans (Albayram et al., 2022).

Choroid plexus epithelium (CPE) also undergoes several senescent changes, which may contribute to inflammaging. Alterations in the CPE cytokine and chemokine profile, such as an increased IL-4:IFN-γ ratio, may contribute to a pro-inflammatory state by upregulation of CCL11, which has been implicated in age-related cognitive decline in murine models (Baruch et al., 2013). Likewise, reduced expression of the transmembrane protein Klotho by the CPE leads to increased MHC II expression, microglial activation, and infiltration by peripheral macrophages (Zhu et al., 2018).

## 3 Immunosenescence in multiple sclerosis

Several immunosenescent phenomena have been observed prematurely in patients with MS, including thymic involution (Duszczyszyn et al., 2010), increased late-differentiated CD8^+^ memory T cells (Haegele et al., 2007), expansion of ABCs (Claes et al., 2016), and somatic telomere length shortening (Guan et al., 2015). Premature immunosenescence in MS may be caused by repeated antigen exposure leading to chronic immune activation, senescent immune cell endurance, and genotypes that enhance primary differentiation (Bolton and Smith, 2018). Evidence of inflammatory synaptopathy, impairment of synaptic plasticity, and altered microglial response to demyelination has also been shown in the brains of older individuals with MS (Musella et al., 2018) and aged mice (Klein et al., 2018), which may contribute to neurodegeneration (Klein et al., 2018; Musella et al., 2018).

Impaired remyelination with advancing age also contributes to inflammaging. This is, in part, due to a decline in maturation of oligodendrocyte precursor cells (OPCs) (Rist and Franklin, 2008). Additionally, aging macrophages have been shown to exhibit reduced efficiency in the clearance of myelin debris in murine models of demyelination (Natra et al., 2015).

Some changes with DMTs may induce changes to the immune system repertoire that are similar to immunosenescence (Mills and Mao-Draayer, 2018). Most other changes with DMTs have anti-inflammatory effects. Fingolimod and glatiramer were shown to have protective effects on rodent microglia (Pul et al., 2011; Noda et al., 2013). In a recent human study, dimethyl fumarate (DMF) decreased the activation and iron content of microglia *in vitro* and reduced susceptibility in rim lesions on magnetic resonance imaging (Zinger et al., 2022).

## 4 Clinical significances of aging in multiple sclerosis

Evidence suggests that the pathogenesis of MS is typified by two concurrent yet distinct inflammatory processes. The first is the invasion of CD8^+^ T cells and CD20^+^ B-cells into the CNS, which is associated with blood-brain barrier breakdown, acute demyelination, and clinical relapse. The second is a slow accumulation of late-differentiated T- and B-cells affecting meninges and large periventricular spaces. This latter process is the predominant driver of inflammaging. However, instead of a direct T- or B-cell immune response, it is the downstream effect of microglia and macrophage activation, oxidative injury, and mitochondrial damage, which contribute to neuroinflammatory tissue injury, neurodegeneration, and disability progression and thus could be relevant therapeutic targets for progressive MS (Lassmann, 2018).

The age-dependent nature of relapse rates affected by inflammation and disability progression in MS supports the hypothesis that mechanisms of immune system aging contribute to disease progression (Tremlett et al., 2008; von Wyl et al., 2020). A cohort study from 2008 suggests that annualized MS relapse rate declines after the third decade of life, even when accounting for DMT use (Tremlett et al., 2008). A subsequent study of 9,705 patients with MS, including 236 pediatric-onset patients with MS, further demonstrates that relapse risk was highest in childhood and decreases continuously to about 35 years of age, stabilizes for about a decade, then subsequently decreases once again. In contrast, disability catalysts remained stable from childhood to age 32 and then increased rapidly around age 45 (von Wyl et al., 2020). This observed decline in relapse rate with advancing age does appear to temporally coincide with the age in which immunosenescent changes naturally occur (Rist and Franklin, 2008). The onset of clinical progression depends more on age rather than disease duration, with progression onset at age 45, controlling for SPMS, single attack progressive MS, and PPMS (Tutuncu et al., 2013). Immunosenescent processes, most predominant with increasing age and an altered inflammatory landscape, may contribute to the annual decline of relapse, which requires a modified therapeutic approach.

Epigenetic changes to immune cell subsets occur gradually, but discrete periods of accelerated change have been identified. The first period occurs between 30 and 40 years, while the second typically occurs in the 60s for men and in the 70s for women (Márquez et al., 2020). The first period may influence relapse risk, while the second drives progression. There is evidence that patients with MS experience altered and potentially accelerated immune cell aging (Theodoropoulou et al., 2019; Hecker et al., 2021). Regarding sex, men display accelerated immune aging, possibly accounting for characteristic aggressive disease progression (Moretto et al., 2008; Jergović et al., 2018). In terms of immune cell subsets, women tend to maintain CD4^+^ T-cell function longer than men, whereas men show a higher degree of epigenetic changes in B cells indicative of a decline or change in function (Márquez et al., 2020). Studies using Siponimod in progressive MS populations suggest that B-cells may be involved in progression (Wu et al., 2020) via epigenetically driven immunological aging (Márquez et al., 2020) as the clinical efficacy of Siponimod may be explained by the shift toward an anti-inflammatory and suppressive homeostatic state (Wu et al., 2020).

## 5 Disease-modifying therapies in older patients with multiple sclerosis

In progressive MS trials, younger age was associated with positive outcomes of therapeutics targeting the adaptive immune system (Mills et al., 2018). The impact of DMTs on aging, compensatory, inflammatory, and neurodegenerative processes is unclear. Some DMTs may modify microglial cell efficacy (Wesselingh et al., 2019), but their effect on innate immune system inflammaging and disease progression is uncertain. Clinical trials commonly exclude older patients, leading to a lack of understanding of the impact of DMTs on age-related immune changes.

To maximize the therapeutic effect and minimize adverse effects, both animal and human trials sample from a specific age range, usually less than 55 years in humans (Mills et al., 2018; Schweitzer et al., 2019; Zeydan and Kantarci, 2020), which can magnify the effect of suppression on active inflammatory demyelination. In progressive MS trials, the primary benefit demonstrated is the reduction of inflammatory relapse, with only a few trials showing a benefit in disability progression (Mills et al., 2018). With the number of elderly patients with MS increasing (Marrie et al., 2010; Daltrozzo et al., 2018), applying study results to the older population is both necessary and challenging. A meta-analysis of clinical trials showed an age-dependent decline in the effectiveness of DMTs and a higher incidence of adverse effects in the older population (Weideman et al., 2017; Vollmer et al., 2021), and it is known that relapse rates also follow an age-dependent decline, suggesting that, in spite of inflammaging, inflammatory demyelination may be less active in the elderly due to immunosenescent changes.

Discontinuation of DMTs in older patients is challenging, and there is no consensus on timing. Early studies, which included younger patients, demonstrated a high relapse rate after DMT discontinuation (Kister et al., 2016; Bsteh et al., 2017). A small study cohort demonstrated that almost 90% of older patients with MS who had no evidence of active inflammation for two years or more had no clinical relapse after one year of DMT discontinuation. In contrast, a high relapse rate was seen in younger patients who had active CNS inflammation within two years prior to discontinuation (Birnbaum, 2017). In another cohort study investigating 600 patients with MS over the age of 60, the vast majority of 178 patients who discontinued DMT remained off treatment, with only one relapse (0.6%), three radiologic progressions (1.2%) (Hua et al., 2019a), and minimal effect on patient-reported outcomes (Hua et al., 2019b). However, most patients included in these studies used either interferons or glatiramer acetate prior to discontinuation. Therefore, this data should be interpreted with caution in patients using highly effective DMTs. Another important consideration in clinical trials using DMTs with immunosuppressive effects is the increased risk of infections seen in the elderly and with accumulated disability (Vollmer et al., 2021; Brand et al., 2022).

A recent clinical trial evaluated the effects of DMT discontinuation in patients aged 55 or older (ClinicalTrials.gov Identifier: NCT03073603). Although it could not be definitively concluded that discontinuing treatment was non-inferior to continuing, a salient takeaway is that new clinical relapses and new radiographic lesions were rare in both study groups (Corboy et al., 2023). There is now an ongoing extension of the DISCOMS study which will provide further information about the safety of DMT discontinuation in older populations.

## 6 Aging and infections in multiple sclerosis

Progressive multifocal leukoencephalopathy (PML) is a severe adverse effect of DMTs, particularly natalizumab, for MS (Warnke et al., 2015). Older age may predict a higher risk of PML development and severe outcomes (Jordan et al., 2020). Although uncommon, PML can occur in older adults without immunosuppression or other comorbidities, causing an immunocompromised state (Chang et al., 2007; Zucker and Stacpoole, 2018) and worse outcomes (Havla et al., 2016; Förster et al., 2019). In an Italian cohort, PML occurred after minimal infusions involving older patients (Prosperini et al., 2016). The risks of HSV1 and VZV reactivation, as well as malignancy, potentially increased with age and DMT use (Schweitzer et al., 2019), likely due to immunosenescence. Several cohort studies investigating the outcomes of COVID-19 in patients with MS showed that older age was associated with greater severity and inferior outcomes (Chaudhry et al., 2020; Louapre et al., 2020; Salter et al., 2021; Simpson-Yap et al., 2021). Specific B-cell depleting therapies (i.e., rituximab and ocrelizumab) were also independently associated with worse outcomes when compared to other DMTs. Therefore, B-cell depleting therapies should be used with caution in older patients with MS during COVID-19 pandemic.

## 7 Aging and vaccine efficacy in multiple sclerosis

Older age is associated with an impaired immune response to primary and booster vaccinations. T-cells favor the generation of short-lived effectors over memory cells (Gustafson et al., 2020), and B-cell response to new antigens is impaired in the elderly, as discussed in Section 2.1. Current evidence suggests that B-cell depleting therapies (Bar-Or et al., 2020) and sphingosine-1-phosphate modulators (Kappos et al., 2015; Ufer et al., 2017; Olberg et al., 2018) significantly blunt humoral immune responses to several vaccines (Ciotti et al., 2020), including influenza, tetanus toxoid (TT), and pneumococcal vaccine. Alemtuzumab may blunt the immune response to vaccines in the first six months after infusion, while the response to prior vaccinations is maintained following alemtuzumab therapy (McCarthy et al., 2013). The above studies did not differentiate between impaired vaccine responses related to disease-specific factors, DMT treatment or aging effect. A few studies demonstrated that natalizumab compromises response to vaccinations, although natalizumab is not known to suppress the systemic immune response (Olberg et al., 2014; Olberg et al., 2018; Metze et al., 2019). In an observational study (Olberg et al., 2014), patients with MS on glatiramer acetate had a lower rate of seroprotection after the 2009 H1N1 “swine flu” vaccination compared to healthy controls. This finding was not reproduced in subsequent studies that used seasonal influenza vaccines (Olberg et al., 2018; Metze et al., 2019). Age, duration of disease, current and prior DMT use may all influence vaccine response (Metze et al., 2019). A randomized control study that compared antibody response to rabies vaccine in healthy individuals who took teriflunomide versus placebo demonstrated that the rabies antibody response was lower in the teriflunomide group. However, all subjects with teriflunomide achieved seroprotective antibody levels (Bar-Or et al., 2015). Several studies have shown that DMF (von Hehn et al., 2018) and beta-interferons do not reduce the immune response to vaccinations (Olberg et al., 2014; Olberg et al., 2018; Metze et al., 2019). In a cohort study investigating the immune response to the hepatitis B virus (HBV), vaccination in older patients with MS was associated with lower antibodies against the HBV surface antigen level following HBV immunization (Faustino et al., 2021).

Regarding COVID-19 vaccines, mRNA vaccines (BNT162b2 and mRNA-1273) have demonstrated high efficacy and immunogenicity. The Ad26.COV2.S vector vaccine was designed as a single-dose vaccine to improve compliance, particularly for older people. This single dose of vaccine resulted in a strong humoral immune response independent of age. Another vector vaccine (ChAdOx1 nCoV-19) needs further investigation to determine the effect of age on immune responses (Teo, 2021). In patients with MS on DMTs, the humoral immune response to the COVID-19 vaccine was reported to be age-independent (Ali et al., 2021). The study also showed that older age, male sex and active smoking were significantly associated with lower antibody titers against SARS-CoV-2 (Pitzalis et al., 2021).Research in this evolving field will help ascertain the aging effects of the COVID-19 and other vaccines.

## 8 Aging and cancer risk in multiple sclerosis

Older age is one of the most important risk factors for the development of cancer, and there is evidence that immunosenescent changes are necessary to promote tumorigenesis. Several changes in the innate and adaptive immune response described earlier in this paper, which occur in the natural aging process, are also induced by the tumor microenvironment. These include a loss of T cell CD28 expression, increased expression of program death ligand 1 by dendritic cells, and a reduction in pro-inflammatory cytokines, all of which contribute to an immunosuppressive state (Ye et al., 2013). Dysfunction of NK-cell-mediated cytotoxicity and a reduction in naive NK cells can also occur in the elderly and has been suggested to play a role in the development of malignancy (Waldhauer and Steinle, 2008).

Autoimmune conditions and cancer are both a result of immune dysregulation. However, there remains conflicting research regarding whether patients with multiple sclerosis have protective effects against cancer due to increased immunosurveillance or whether they are at increased risk due to chronic inflammation. A higher total incidence of malignancy, as well as an increase in malignancy subtypes (e.g., colorectal, prostate, breast), among MS patients compared to the general population was suggested in one French cohort (Bosco-Levy et al., 2022). Regarding the associated risk among elderly MS patients, an Italian cohort found an increased risk of malignancy in the subpopulation of women with MS over the age of 50, although there was no significant difference between malignancy incidence overall compared to the general population (D'Amico et al., 2019). Likewise, an increase in breast cancer among postmenopausal women with MS compared to age-matched controls was observed in Sweden, whereas there was no increased incidence among premenopausal women (Hajiebrahimi et al., 2016). In contrast, reduced malignancy incidence was observed in cohorts in British Columbia (Kingwell et al., 2012) and New York (Gaindh et al., 2016).

Additionally, the initiation of DMTs for treatment may lead to further immunomodulation or immunosuppression, increasing cancer risk (Bahmanyar et al., 2009). In prior clinical trials, interferons and glatiramer acetate have not been shown to increase the incidence of cancer, unlike natalizumab (Polman et al., 2006), fingolimod (Cohen et al., 2010; Comi et al., 2010; Cala et al., 2014; Lublin et al., 2016), siponimod (Kappos et al., 2018), teriflunomide (O'Connor et al., 2011; Vermersch et al., 2014), cladribine (G et al., 2010), alemtuzumab (Coles et al., 2017; Havrdova et al., 2017), rituximab (Salzer et al., 2016), ocrelizumab (Hauser et al., 2017; Montalban et al., 2017), and DMF (Fox et al., 2012; Gold et al., 2012). Additionally, some DMTs, such as rituximab and cladribine, have been used as cancer treatment prior to their utility in MS. Therefore, there remains a complex interplay between cancer risk and multiple sclerosis and treatment. Prior studies have shown that increased cancer risk in patients on azathioprine, methotrexate, cyclophosphamide, and mitoxantrone is partially attributed to the family history of cancer and cumulative dosage and duration of their treatment (Lebrun et al., 2011; Ragonese et al., 2017).

## 9 Knowledge gap

Aging is associated with immunosenescence, but the specific changes relevant to disease progression in MS are not well established. The timing of a future progression-targeted therapeutic intervention is crucial and may require non-invasive biomarkers for detection. Biological age measured by leukocyte telomere length (Krysko et al., 2019) and ovarian age measured by the anti-Mullerian hormone is associated with disability progression in MS (Graves et al., 2018). However, the impact of ovarian aging and sex hormones on immunosenescence is not well understood.

Although some DMTs, such as fingolimod (Noda et al., 2013) and glatiramer acetate (Pul et al., 2011), show effects on microglia in animal models, their clinical benefit in human progressive MS has not been proven. Further studies are needed to understand why these effects have not translated to clinical benefit. DMF has shown promising results in reducing microglia activation and iron content on susceptibility MRI (Zinger et al., 2022), but more validation is needed for its use as an imaging marker. It is important to investigate DMTs targeting senescent and chronically activated innate immune cells and their effects on nonactive progressive MS (Mills and Mao-Draayer, 2018). Rejuvenation strategies targeting various immune system components have been proposed, including hematopoietic stem cells, oligodendrocyte precursor cells, microglia, monocytes, thymus, and senescent lymphocytes (Dema et al., 2021).

## 10 Conclusion

Immunosenescence is an age-related loss of innate and adaptive immune system proficiencies, which influences the course of and accelerates MS. Thus, senescent immune cells may play an essential role in progressive MS. Some DMTs may induce similar immune system changes. Age-related changes in the immune system may contribute to the risk of adverse events, particularly infection, in older patients with MS. However, none of the available DMTs effectively target the immunosenescence process. Therefore, the development of treatment strategies to rejuvenate the immune system may be of hope for patients with progressive MS.
